# Shock, in Relation to Dental Operations

**Published:** 1889-01

**Authors:** James Truman


### International Dental Journal.

Vol. X. January, 1889. No. 1.

### Original Communications.[1]

### SHOCK, IN RELATION TO DENTAL OPERATIONS.[2]

BY JAMES TRUMAN, D.D.S.

I am about to treat of a matter which I am well aware is not usually considered within the domain of the dental operator, and yet one familiar in all its phases to the medical observer. I am, therefore, placed in the rather unenviable position of taking a theme not of general interest to the dental practitioner, and, while not fully understood, somewhat trite to the general medical thought. My object is, however, to lead up to inferences from facts in general practice to, perhaps, a more intelligent conception of certain phenomena observable in dental practice. In order to do this, I am forced to the consideration of shock, or collapse, as a condition preliminary to my deductions; in a word, I desire to accentuate the known facts in order that I may draw conclusions to be made more or less obvious as I proceed.

It is a well-known fact that death may come to the individual and no trace of antecedent injury be manifest. Depression to the general circulating system may be apparent, and the medical attendant be wholly at a loss to define the cause. Mental emotion may produce changes at once rapid in its effects and leaving results of a character that time may scarcely efface. The true definition of shock may be termed a "sudden depression of the vital powers resulting from an injury, more or less grave, or an impression made

[1] The editor and publishers are not responsible for the views of authors of papers published in this department, nor for any claim to novelty, or otherwise, that may be made by them. No papers will be received for this department that have appeared in any other journal published in this country. The journal is issued promptly on the 15th of the month.

[2] Read before the Odontological Society of Pennsylvania, November 3, 1888.

on the nervous system, or by fright; sudden and overpowering mental emotion." (Black.) While death may result from such a depression of vital powers, this extreme result is by no means always the case; but that the changed condition of the circulation may lead up to grave symptoms is clear, when it is understood that the delayed phenomena may be more serious than would be suggested at the earlier and more active stage of shock.

To judge of the subject intelligently, the origin of collapse will be considered as briefly as may be consistent with the importance of the subject. Shock, or collapse, must be regarded as arising from an altered condition of the circulation produced by direct or reflex action on the nerve-centres, and without leaving any evidences of "change in the tissues; but while this is unquestionably the case in some instances, there may be in others post-mortem evidences of morbid effects. The view that shock is always dependent on an altered state of the nerves has been combatted, and Jordan has shown that potassium cyanide, by acting directly on the cardiac muscular fibre, and by impairing its contractility, gives rise to those numerous secondary effects of shock which depend on an arrested or imperfect supply of arterial blood." The condition of knowledge in regard to the action of the vaso-motor system of nerves is, perhaps, too imperfect to assign positive reasons or to argue the question absolutely from facts. Sufficient is known, however, to form a basis of reasonable inference for much of the phenomena observed. These so certainly point to nerve influence on circulation that the conclusion is inevitable,—that the impulse proceeds from a centre or centres of nerve action, and the circulation is changed by a direct loss of tonus in the vessels. That this may be understood, permit me to refer in a few words to the prevalent theories in regard to the action of the nerves on the circulation. "The dependence of the vessels upon the nerves consists in this: that the latter keep the muscular fibre of the arteries in constant tonic contraction, so that an active resistance is opposed to the expansion of the vessels from blood pressure. The continuous excitation upon the arterial wall is exercised by the sympathetic. After section of this nerve, in the neck, the lateral pressure of the walls of the vessels instantly diminishes. The blood in proportion to the pressure dilates the arteries, and further on the capillaries of the corresponding side of the head, and runs through these with such velocity that, since nutrition does not increase with the growth of the blood, it does not become venous, but remains arterial, and that, besides, since the resistance of the arteries is destroyed, the pulse continues into the capillaries, and even into the veins; in consequence, there occurs reddening and an elevation of temperature from 3°–6° C. On the other hand, irritation of the superior cervical ganglion is followed, but more slowly, by contraction of the same vessels, paleness, and lowering of the temperature." (Wagner.) The excito-motor centre of these vaso-motor nerves is admitted to be the medulla oblongata, and "the spinal cord also is throughout an independent central organ for the vaso-motor nerves." (Goltz.) Dr. Marshall Hall (1831) made many experiments to demonstrate the effect of the brain and cord upon the circulation. He says: "It is quite obvious from these experiments that the circulation no more depends upon the medulla oblongata than upon the medulla spinalis." Without entering upon an extended examination of theories, based though they may be on facts, the generally recognized view now is that the circulation is affected by direct irritation and by reflex action, and that "special vaso-motor nerve-centres exist for the various vascular provinces." (Wagner.) Section of a vascular nerve will produce, therefore, a flow of blood to the parts to which it is distributed; that excitation by the interrupted current, or by mechanical means, produces constriction of the minute arteries; that excitation of a sensory nerve produces increased activity of the capillary circulation." (Simon.) The heart will continue to act after the removal of the nerve-centres, and hence is not directly dependent upon these, and yet it is clear that mental emotions have a direct and powerful influence upon this organ sufficient, in many cases, to produce death. True shock, as defined by some writers, must be limited to its "immediate production," while others attempt to classify it as "transitory, delayed, protracted, and insidious."

Without entering into the discussion in regard to the probability of other factors having a place in the question of shock, such as fat embolism, fat cells being absorbed from "fractured bones or lacerated adipose tissue;" or whether the continued depression is to be referred to direct or reflex action on the nerve centres, there remains this fact, that collapse is produced through a change in the circulation.

In the slighter degrees of collapse, the patient may present no marked symptoms, makes no complaint and experiences no pain. The extremities are cold, face more or less exhibiting a pinched expression. From this it may pass to the extreme form, with pallor on the surface, lips pale and bloodless, motionless, cold over the body, hardly perceptible pulse, great weakness, oppression, dizziness, nausea, confused perceptions, and respiratory movements feeble. These symptoms are not always confined to severe cases of physical injury or to excessive mental emotion; but will be manifest oftentimes after most trivial injuries, the effect being out of all proportion to the cause.

The relations which the phenomena of shock bear to dental operations may not be clear to the average observer; but to my mind they embody much subject for serious thought, and ought to lead to a clearer apprehension of our duty as practitioners.

While the evidence is very far from absolute that the conditions we are familiar with are dependent for a solution on shock, yet they are so closely allied to the phenomena already adverted to that one is naturally drawn to inferences and suggestions. It is unnecessary, in my judgment, to confine our observations to the extreme cases of collapse, for, as I have before stated, the symptoms will be very variable. The mental emotion caused by the sudden loss of near and dear friends may not amount to shock in the extreme sense; but who has not observed the long periods of weakness, the lack of mental force, the general loss of tone in the circulation, which may take months, and even years, to recover from? Whether this be ascribed to continued shock or to other pathological sequences, it certainly had its origin in a deep impression made on the nervous system, and, consequently, in a loss of controlling power. My observations and conclusions lead me to the opinion that these phenomena must be ascribed to a modified form of collapse. The mental strain produced in times of great public excitement—the effect upon a merchant who has ended a carefully ordered life with failure in business—the rapid decline of those who have commanded large bodies of men in war through many battles, most noticeable since the Rebellion—these all showing a nervous strain, and producing symptoms and lesions which must be ascribed to the insidious working of nerve influence.

It seems to me impossible to avoid the conclusion that many serious conditions, now unexplained, must be attributed to this cause; at least many more than are now generally recognized. What is weariness but a similar effect? We call it shock when the impression is a powerful one; but is not this only a form of insidious impression, an action on the nerve centres in response to peripheral sensations? Make this an over-strain, and, if repeated and repeated, the tonus of the vessels is lost altogether, and the individual succumbs. This may not be collapse in a scientific view; but is certainly an approach, and closely allied to it in the broader sense that the greater includes the less, and cannot be explained intelligently in any other way.

To apply these thoughts to dental operations and dental operators is a natural sequence. Dr. Black, in "The System of Dentistry," has made most of these facts familiar to you, and his views on the overtaxing of patients should be carefully read and pondered. He illustrates the importance of attending to this matter by a case in his own practice, and, as this bears directly on the subject, I quote it in full:

"A young lady of eighteen came from a distance by appointment to have carious teeth filled. Upon examination it was found that there were two exposed pulps, besides other smaller cavities. Both the young lady and her parents insisted that all should be done that day if it were possible. The operations were proceeded with, and everything without a murmur. My patient was a fine specimen of physical development, and I soon found that she prided herself on her powers of endurance. The pulps were, at her urgent request, that there should be no delay, removed directly with the broach, and the filling proceeded with. After three hours of continuous operation, the patient was discharged for two hours' rest. She returned promptly, but something in her appearance arrested my attention as not being just right; yet, in answer to questions, she said she felt perfectly well, only a little tired. The operations were resumed, and all went well at first; but after an hour, the latter part of which had been occupied in the excavation of a very sensitive cavity, I found the pulse had become very compressible, and other evidences of shock were becoming apparent. Gutta-percha fillings were placed in the cavities excavated, and operations suspended. I found it necessary to assist her to a couch, as it was evident that she was unable to walk steadily. After two hours in the recumbent posture she seemed better, and was taken to the train by her parents, and I saw her no more. I afterwards learned from her mother that her condition became much worse *en route* home, and that for four or five days she was in a "stupid condition," and after this she passed into a nervous fever which continued for several months. Up to the time I last heard from her, four years after the incident, she had been more or less an invalid."

Now this case may be, and is unquestionably, an extreme one; but we all know of cases, and by no means infrequent, of persons exhibiting great weakness and depression after prolonged dental operations. Indeed, so common has this been with me, that for years I have been unwilling to extend the sitting over two hours, and then to insist on an intermission of several days. We, as dentists, interested in the operation at hand, can scarcely realize the nervous tension to which our patients are subjected. This mental and physical strain is sure to produce a condition of collapse, modified in extent though it may be, still, by constant repetition, may produce results of a grave character. The very long operations of from six to seven hours, and even longer, are, it is hoped, passed by. The craze for enormous gold operations has not only in the past depleted the purses of our patients, but has had equally injurious results upon the circulatory system. While such operations may truthfully be defended on the score of value to the teeth, they cannot be recommended in view of the possibility of permanent injury to the individual.

Surgical shock may meet us directly in cases of extraction. The shortness of this operation may lead some to regard it as of little moment; but there is probably no operation that the average mind will not more calmly consider than this. A few favored individuals blessed with very " strong nerves " can sit down and have a tooth out with a nonchalence surprising; but these are exceptional. With most of us it is a great mental strain in advance, and a great shock during the operation, and the consequent depression is fully in accord with what we know of the phenomena accompanying more important surgical cases. It has been too much the custom, in the past, to remove teeth as long as the " patient will stand it;" or, in other words, until the sufferer refuses longer to allow the forceps to enter the mouth. Hence, it is not unusual for persons to have twenty or even twenty-four teeth removed at one sitting, and dentists have been known to pride themselves on their agility in handling that many teeth in a given time. If my views, as heretofore stated, be considered as having any force, are not such operators guilty of malpractice? They certainly have taken risks that may reach beyond the limits of endurance, and, should untoward results follow, they are to blame. I presume all who listen to me to-night, of the older circle, will recall cases, in their ignorance—and we have all been ignorantly guilty in this—where, after many extractions, the patient has been confined to the bed for days with all the symptoms I have detailed. It was from such experiences that I was led in years past to refuse to extract more than six teeth at one sitting. This I regard as the only safe practice, from this point of view as well as from that other, more remote, liability to hemorrhage.

I have alluded to two forms of injury through dental operations. These might be multiplied; but they will suffice to call attention, I trust forcibly, to the necessity of great care that lasting injury be not produced in serious lesions to various organs, through this lapsing into continuous shock.

There is, however, another branch of my subject that I regard as of more importance, because it interests ourselves, and while this may be and is a somewhat selfish view, we must meet it, not only for our own good, but for the good of future generations of patients. The profession of dentistry is certainly one of the most trying at present organized into a calling. This remark I know will be met with the assertion that the exacting labors and anxieties of the physician, and the mental strain of the surgeon in capital operations, furnish evidence of greater and more prolonged nervous tension. Without attempting to argue this point, for it must be admitted that in exceptional cases the strain is greater, the fact must be recognized that the dentist, from the nature of his occupation, cannot have any respite during the working hours of the day. This means more than mere labor; it is an unbroken effort of the mental and physical powers of the operator in almost constant conflict with similar disturbed relations of the patient. The interchange of psychical influences, under such circumstances, is but little understood; but the fact is well known that one nervous patient will exhaust the operator more in one hour than a day's work over those of a quieter character. Standing, as most do, from eight or nine o'clock in the morning until four or five in the afternoon, without proper food, perhaps a glass of milk hastily swallowed, or entire abstinence, and day after day meeting all sorts of conditions of men, women, and children, the women all nerves, and the men all impatience, and the children all frightened, what, I ask, is to be expected? A few have the happy faculty of so ordering their work that it seems to leave no bad results; but the average man cannot do this, and he arrives at the end of each day with the mental and physical organization in torture. The pinched expression of his face, the sunken look about the eyes, the intense fatigue, are indicative of great nervous depression, and point directly to a minor manifestation of shock. Let this be continued day by day, month by month, and how many years do you suppose it will be before the over-strained nerves will yield, and fail to perform their proper function in regulating the circulation by keeping up the tonus of the arterial system?

As I look back over many years spent in professional work, I cannot help recalling many familiar faces that have passed away, all too early, from their active work. As I marshal them before me, I remember bright young lives wrecked before they had scarcely begun to develop. This, while undoubtedly true of all callings, has, it seems to me, more notable cases in ours. When it is observed that the depressed condition I have referred to eventually may end in centres of inflammation in important organs—lesions that do not explain themselves, an everlasting feeling of weariness that never finds rest—all directly traceable to the continuous strain of routine professional work, it is time to pause. Is the picture overdrawn? I think not. The fact is familiar to all of you, and, doubtless, comes nearer home to many. What does it mean when the dentist throws himself down at the close of his day and, as he expresses it, is "utterly worn out?" What does it mean that, month by month, he feels less and less able to perform the routine work of the office? What does it mean when, in after years, he may be forced to seek other occupation, or yield up a portion of his specialty? What does it mean that our profession is largely made up of young men? We are familiar with the old doctor, with the old lawyer, with the old merchant; but the old dentists are so few that one is tempted to ask, "The fathers; where are they?" Examples are so numerous of this condition among dentists, that I would simply weary you with the telling; and I will, therefore, give one or two as illustrations. My mind reverts to one very familiar to me in former years. From eight in the morning until eight in the evening, of the long days of summer, he stood closely by his chair. Having partaken of a late dinner, he would throw himself down on the lounge, and lie as one in a profound collapse. This was the almost unvarying habit of years. His periods of relaxation were short and far between. He died at middle life of paralysis. Another I recall with a large practice. He never knew when to stop. His friends exhausted their powers of persuasion, myself among the number, without result. In time he too came to be a wreck, and shortly thereafter passed on into the unseen. But why multiply cases; you understand as well as I that the primal law of the organization is that there is a limit to the powers of all the organs of the body, and that activity and rest are alike essential to healthy growth; but strain means weakness, and weakness means ultimate death.

The effect of anesthetics in producing shock has not been alluded to, except incidentally, by any writer that I am aware of; but I am convinced that they furnish the conditions and produce results that simulate shock very closely. I am satisfied from my observations on persons and myself that all the symptoms of true shock may be present on the return of consciousness, and that this may last for days. The patients complain of great weakness; the face is pale, indicative of defective circulation, and the general appearance simulates collapse. Some years ago I had nitrous-oxide administered to myself by one skilled in its use. Though quite familiar with the administration of anesthetics, I had never inhaled this agent, and commenced the inflation of the lungs with determination to watch the physical effects if possible. The result was a profound narcosis as far as the body was concerned; but a very acute ability to analyze all sensations mentally. At no time were the reasoning powers obscured; nor was the extraction felt physically. While there were some curious psychic effects observed in this operation, the point that impressed me the most was the subsequent influence upon the organization. The shock was so marked that for two days afterward I found my strength was inadequate to the slightest exertion, and it was weeks before the normal condition was resumed. The personal experiences of an accident, some years previous, followed by collapse, were vividly recalled; as they were the same in kind, if not in degree.

Prof. C. N. Pierce, quoted by Dr. Litch (System of Dentistry), writing of the case of a Dr. K—— who had a number of roots extracted under the influence of nitrous oxide, says of him that he was a man of good health, weighing two hundred pounds. After the extraction he went to his place of residence, distant but a few blocks, where he remained for some hours, feeling very much prostrated, his face having an ash color, and his circulation not resuming its normal condition for some hours. Within ninety days he lost forty pounds in weight, which was never recovered. He eventually died of disease of the kidneys. I am perfectly aware that this condition is ascribed to the asphyxiation of the blood by nitrous oxide, and that one authority asserts "that during asphyxia there is produced a veritable shower of sugar in the blood," producing diabetes, or aggravation of that disease if present. While this is probably true, it does not explain my own case or that of many others.

The limits of a paper will not admit of an extended examination of this or those heretofore adverted to. They are, as stated at the beginning, only suggestions, which, I hope, may lead to thoughtful consideration.

